# *Salvia miltiorrhiza* Induces Tonic Contraction of the Lower Esophageal Sphincter in Rats via Activation of Extracellular Ca^2+^ Influx

**DOI:** 10.3390/molecules200814504

**Published:** 2015-08-11

**Authors:** Ching-Chung Tsai, Li-Ching Chang, Shih-Che Huang, Shu-Leei Tey, Wen-Li Hsu, Yu-Tsun Su, Ching-Wen Liu, Tong-Rong Tsai

**Affiliations:** 1 School of Pharmacy, Kaohsiung Medical University, No. 100, Shih-Chuan 1st Road, Sanmin District, Kaohsiung City 807, Taiwan; E-Mails: u101130@yahoo.com.tw (C.-C.T.) ; fruit0227@hotmail.com (C.-W.L.); 2 Departments of Pediatrics, E-Da Hospital, I-Shou University, No. 1, Yida Road, Jiaosu Village, Yanchao District, Kaohsiung City 824, Taiwan; E-Mails: djsr2000@hotmail.com (S.-L.T.); suyutsun@yahoo.com.tw (Y.-T.S.); 3 Department of Occupational Therapy, I-Shou University, No. 8, Yida Road, Jiaosu Village, Yanchao District, Kaohsiung City 824, Taiwan; 4 Department of Pharmacy, E-Da Hospital, I-Shou University, No.1, Yida Road, Jiaosu Village, Yanchao District, Kaohsiung City 824, Taiwan; 5 Department of Internal Medicine, E-Da Hospital, I-Shou University, No. 1, Yida Road, Jiaosu Village, Yanchao District, Kaohsiung City 824, Taiwan; E-Mail: shihchehuang@hotmail.com; 6 School of Medicine, I-Shou University, No. 8, Yida Road, Jiaosu Village, Yanchao District, Kaohsiung City, 824, Taiwan; 7 Institute of Basic Medical Sciences, Medical College, National Cheng Kung University, No. 1, Dasyue Road, East District, Tainan City 701, Taiwan; E-Mail: hsuwenli0626@gmail.com

**Keywords:** *Salvia miltiorrhiza*, tonic contraction, lower esophageal sphincter

## Abstract

Up to 40% of patients with gastroesophageal reflux disease (GERD) suffer from proton pump inhibitor refractory GERD but clinically the medications to strengthen the lower esophageal sphincter (LES) to avoid irritating reflux are few in number. This study aimed to examine whether *Salvia miltiorrhiza* (SM) extracts induce tonic contraction of rat LES *ex vivo* and elucidate the underlying mechanisms. To investigate the mechanism underlying the SM extract-induced contractile effects, rats were pretreated with atropine (a muscarinic receptor antagonist), tetrodotoxin (a sodium channel blocker), nifedipine (a calcium channel blocker), and Ca^2+^-free Krebs-Henseleit solution with ethylene glycol tetraacetic acid (EGTA), followed by administration of cumulative dosages of SM extracts. SM extracts induced dose-related tonic contraction of the LES, which was unaffected by tetrodotoxin, atropine, or nifedipine. However, the SM extract-induced LES contraction was significantly inhibited by Ca^2+^-free Krebs-Henseleit solution with EGTA. Next, SM extracts significantly induce extracellular Ca^2+^ entry into primary LES cells in addition to intracellular Ca^2+^ release and in a dose-response manner. Confocal fluorescence microscopy showed that the SM extracts consistently induced significant extracellular Ca^2+^influx into primary LES cells in a time-dependent manner. In conclusion, SM extracts could induce tonic contraction of LES mainly through the extracellular Ca^2+^ influx pathway.

## 1. Introduction

Gastroesophageal reflux disease (GERD) is caused by reflux of acid or other irritants from the stomach into the esophagus. The main cause of GERD is incompetence of the lower esophageal sphincter (LES) which normally closes at the esophagogastric junction to avoid the back-up of food and acid injury and transiently relaxes to allow food from the esophagus into the stomach when swallowing [1]. Acid reflux-induced mucosal inflammation may affect muscle and nerves, and then affect LES and esophageal body motility. Further acid reflux and mucosal damage may cause a vicious cycle of impaired LES function and esophageal injury. Treatment for GERD includes lifestyle modifications, medications, and surgery. Proton-pump inhibitors (PPI), histamine type II receptor blockers or antacids are common medications for GERD. PPI is the first-line medication for GERD, but 10%–40% patients with GERD are refractory to PPI and furthermore, long term use of PPI may lead to complications [2]. The surgery for severe GERD usually involves strengthening the LES and repairing the hiatus hernia [3]. Clinically, there are few medications, such as baclofen which decreases LES relax and then indirectly increases LES pressure for the patients with PPI refectory GERD. However, baclofen may cause central nervous system side effects [4]. Therefore, treatment of PPI refractory GERD is still a challenge and it is important to develop drugs that increase LES contraction to avoid irritant reflux.

*Salvia miltiorrhiza* Bunge (SM), also known as red sage or Danshen in Chinese, is clinically used in Traditional Chinese Medicines and officially listed by the Pharmacopoeia Commission of the People’s Republic of China [5]. SM is a common drug affecting blood viscosity and its effects include improving blood circulation, removing blood stasis, promoting blood flow in menstruation, resolving mental uneasiness and restlessness, nourishing the blood, tranquilizing the mind, eliminating and breaking stone, treating gurgling in the intestines, relieving fullness, and resolving swelling [6,7]. SM or its active components are widely used as an alternative medicine to treat coronary heart disease, cerebrovascular disease, hepatitis, cirrhosis, chronic renal failure and osteoporosis, and SM has been reported to have anti-cancer effects [8,9,10,11,12].

There are a few studies of SM concerning gastrointestinal motility or disorders to date. The increase in expression of cholecystokinin and vasoactive intestinal peptide in the jejunum was suppressed by SM pretreatment, which might contribute to the early recovery of gastrointestinal motility in digestive tract congestion injury caused by liver ischemia [13]. SM can decrease the inflammation of colon induced by dextran sulfate sodium in rats [14]. SM can ameliorate the pathological changes in the small intestine, spleen and thymus and decrease the mortality rate of rats suffering from severe acute pancreatitis [15]. Our previous study found that SM can induce tonic contraction of the ileal tube in rats through intracellular Ca^2+^ release and the Ca^2+^-calmodulin pathway [16].

To the best of our knowledge, no study about the effects of SM on LES has been reported to date. The aim of this study is to therefore to investigate the contractile effects of SM extracts on LES and the mechanism(s) involved. We found that SM extracts can induce LES contractile responses *ex vivo*. To explore the mechanisms that mediate SM extracts-induced contraction of LES, atropine (a muscarinic receptor antagonist), tetrodotoxin (a neuronal sodium channel blocker), nifedipine (a calcium channel blocker), or Ca^2+^-free Krebs-Henseleit solution with ethylene glycol tetraacetic acid (EGTA) was given and then cumulative dosages of SM were added. Then, the isometric contractions of LES muscle strips were measured in organ baths.

## 2. Results and Discussion

### 2.1. The Content of Tanshinone IIA (TA) in SM Extracts

According to the Pharmacopoeia Commission of the People’s Republic of China [5], TA has been used mainly for quality control of the roots of *S.* *miltiorrhiza Bunge* (Danshen). The content of TA was detected by high performance liquid chromatography (HPLC) and quantified as 1190 μg/mL in SM extracts. The extraction yield of TA from SM roots was calculated as 0.0595%.

### 2.2. Dose-Response Effect of SM Extracts on Contraction of LES

**Figure 1:**
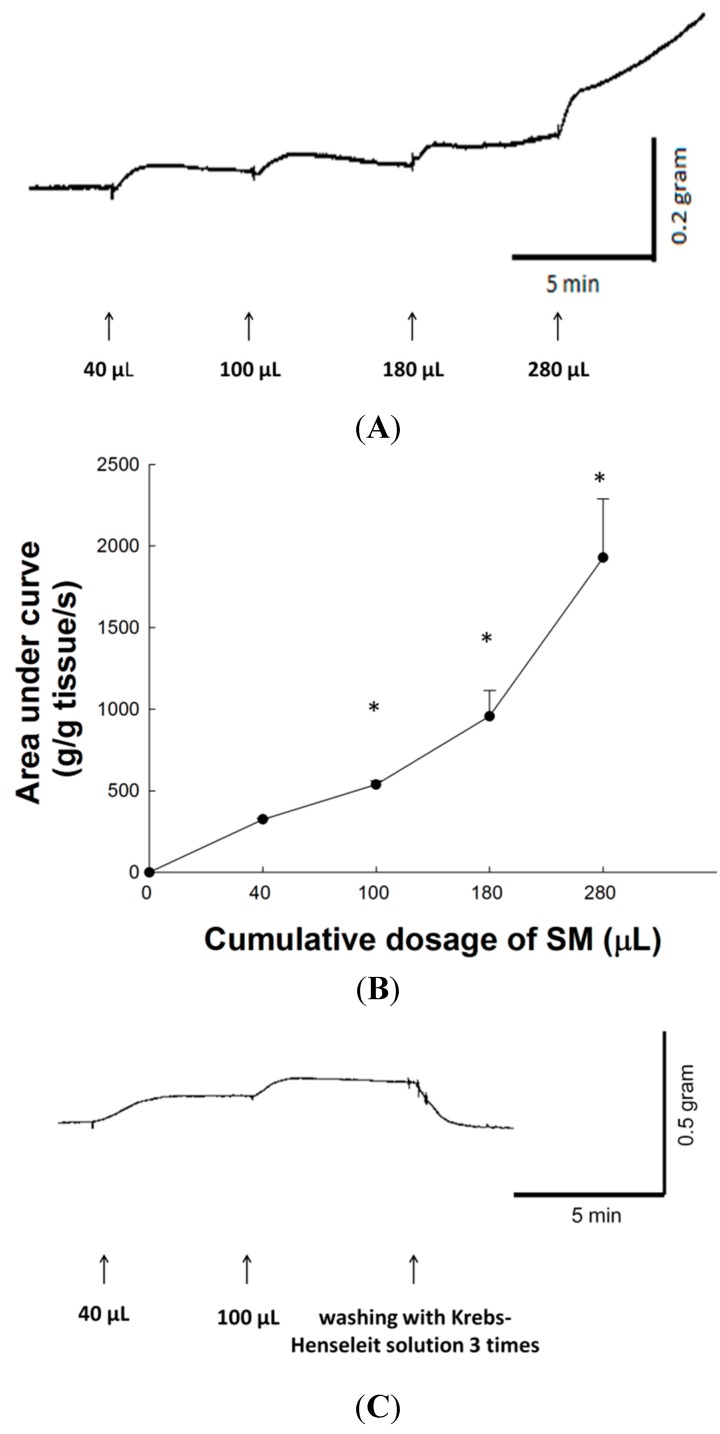
The SM extracts-induced contractions of LES in rat. (**A**) Typical tracing of the contraction of LES in rat in response to cumulative addition of SM extracts; (**B**) Concentration-response curves for the SM extracts-induced contractions of rat LES. Arrows indicate the addition of SM extracts on cumulative dosage at intervals of 5 min; (**C**) Contraction curve of LES returned to prestimulation level after removing SM extracts with Krebs-Henseleit solution. The contractile change was compared to resting contractility of corresponding experiments. * Significantly different from the resting contractility at *p* < 0.05.

SM extracts produced a dose-dependent tonic contraction on isolated LES of rat. Figure 1A shows typical tracings of SM extract-induced tonic contraction of rat LES. Figure 1B shows the contractile responses and area under the contraction curve (AUC) produced in rat LES by the application of increasing concentrations of SM. Compared to the basal line, the AUC on the contraction curves on cumulative dose of 40, 100, 180, and 280 μL SM extracts were 324.8 ± 6.5, 537.8 ± 23.8, 956.3 ± 158.4, and 1927.4 ± 361.7 (g/g of tissue weight/s) respectively. Thus, SM enhanced the contractile activity of LES in a dose-response manner. The LES contraction curves induced by cumulative doses of 40 and 100 μL SM extracts returned to prestimulation levels after removing SM extracts with Krebs-Henseleit solution (Figure 1C). Therefore, the tonic contraction of LES induced by SM extracts was reversible. After removing SM extracts with normal Krebs-Henseleit solution, the LES maximum contraction was remeasured by carbachol addition. The contractile percentage of LES after SM treatment was 96.3% ± 1.1% of pre-SM stimulation level (*p* > 0.05). Thus, the increased tonic contraction of LES induced by SM extracts probably does not happen via toxic effects.

The mechanism controlling the contractile activity of the LES is incompletely understood. It may be regulated or modulated by many factors, such as nerve ending-smooth muscle interactions, neurohumoral substances, diet and drugs [17]. This study identified for the first time that SM extracts can induce tonic contraction of LES in rats. The contraction of the smooth muscle of LES is affected by multiple factors but mainly by autonomic neural innervations. Excitatory postganglionic vagal nervous terminals release acetylcholine (ACh) in the myenteric plexus, resulting in activation of muscarinic (M) receptors. Activation of M receptors increases the intracellular concentration of Ca^2+^. Increased cytosolic Ca^2+^ comes from the extracellular entry through trans-membrane Ca^2+^ channels or/and the release of intracellular Ca^2+^ stores [18]. An increase in cytoplasmic calcium concentration, binding to calmodulin and resulting in Ca^2+^/calmodulin-dependent activation of myosin light chain kinase (MLCK), is an important pathway for contraction of LES. In addition, resting LES tone is related to a protein kinase C-dependent pathway [19]. Various mechanisms may be involved in the SM extract-induced contraction of LES and they are described as follows.

### 2.3. Effect of Tetrodotoxin (TTX) on SM Extracts-Induced Contraction of LES

**Figure 2:**
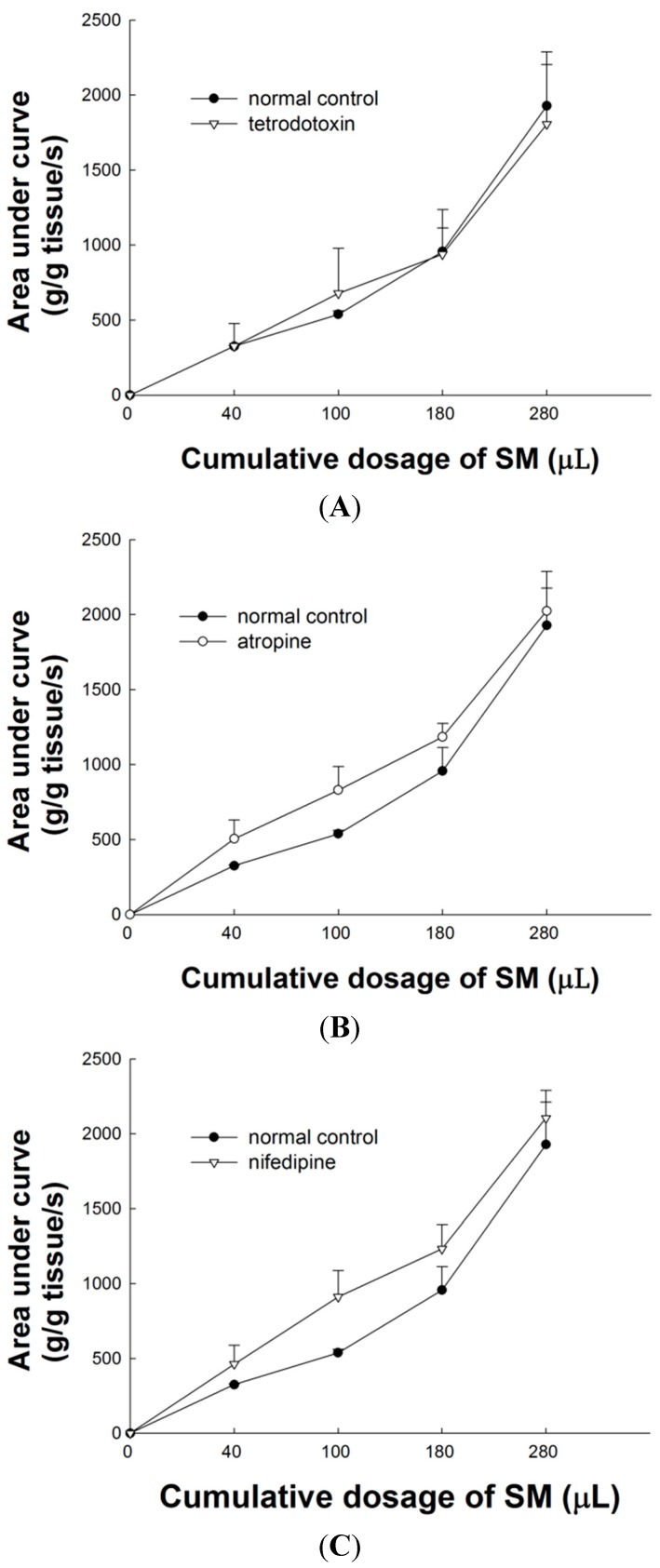
Effects of pretreatment with TTX, atropine and nifedipine on contraction of LES induced by SM extracts. (**A**) TTX (10^−6^ M) has no significant effect on contraction of LES induced by SM extracts; (**B**) Atropine (10^−6^ M) has no significant effect on contraction of LES induced by SM extracts; (**C**) Nifedipine (10^−6^ M) has no significant effect on contraction of LES induced by SM extracts.

TTX has no significant effect on the area under the contraction curve of SM extract-induced contraction of LES. As shown in Figure 2A, the dose-response curves of SM extracts were almost unaffected by 15 min pretreatment with TTX (*p* > 0.05 compared with administration of SM extracts, *n =* 3). The efferent neurons from excitatory vagal nerves have the main regulatory action to increase LES tone. TTX is a selective neuronal Na^+^ channel blocker. To investigate whether SM acts directly on the nerve fiber or on smooth muscle of LES, the effect of TTX (10^−6^ M) on SM-induced tonic contraction of LES was examined in the present study. No significant difference of TTX on SM extracts-induced contraction of LES was observed, compared to control. The result suggests that SM does not act directly on the nerve fibers of LES.

### 2.4. Effect of Atropine on SM Extract-Induced Contraction of LES

The non-selective muscarinic receptor antagonist atropine (10^−6^ M) was also studied its relation to SM extracts-induced contraction of LES. As shown in Figure 2B, 6-min pretreatment with atropine has no significant effect on SM extracts-induced contraction of LES (*p* > 0.05, *n =* 3).

In LES, excitatory postganglionic myenteric neurons, releasing acetylcholine and its derivatives such as carbachol, activate muscarinic receptors, inducing increased cytosolic [Ca^2+^]_i_ and contraction [20]. Muscarinic receptors are divided into five subtypes, M_1_–M_5_. Among them, the M_1_, M_2_ and M_3_ receptors are thought to be the main ones associated with the contraction of LES. Non-selective muscarinic receptor antagonists like atropine can block the M_1–5_ receptors. To understand the mechanism underlying the contractile effects of SM extracts on LES, atropine was used to investigate the possible involvement of the cholinergic pathway. We found that atropine (10^−6^ M) failed to inhibit SM extract-induced contraction of LES, indicating that the SM extract-induced contraction might not be mediated through the muscarinic receptors.

### 2.5. Effect of L-Type Ca^2+^ Channel Blocker on SM Extract-Induced Contraction of LES

The L-type Ca^2+^ channel blocker nifedipine (10^−6^ M) has no significant effect on the area under the contraction curve of SM extract-induced contraction of LES. As shown in Figure 2C, the dose-response curve of SM extracts was almost unaffected by a 20 min pretreatment with nifedipine (*p* > 0.05, *n =* 3). We confirmed that the L-type Ca^2+^ channel blocker, nifedipine, failed to block SM extract-induced contraction of LES.

### 2.6. Effect of Ca^2+^-Free Krebs-Henseleit Solution plus EGTA on SM Extract-Induced Contraction of LES

**Figure 3:**
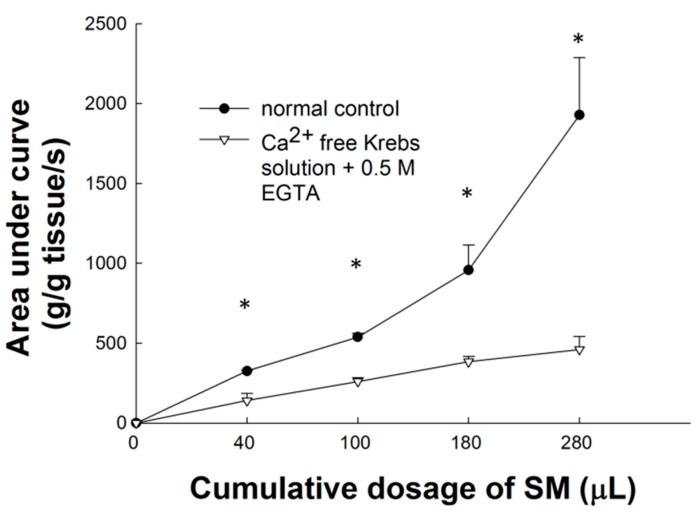
Effects of Ca^2+^-free Krebs-Henseleit solution with EGTA on contraction of induced by SM extracts. Ca^2+^-free Krebs-Henseleit solution with EGTA significantly (*p* < 0.05) inhibited the SM extract-induced contraction of LES in a dose-dependent manner. * Significantly different from the control at *p* < 0.05.

Compared to the basal line, the AUC of the contraction curves after cumulative doses of 40, 100, 180, and 280 μL SM extracts were 140.4 ± 45.6, 260.3 ± 22.3, 383.7 ± 33.8, and 460 ± 82.1 (g/g of tissue weight/s) respectively. Ca^2+^-free Krebs-Henseleit solution plus EGTA has a significant effect on different dosage of SM extract-induced tonic contraction (*p* < 0.05, *n =* 3; Figure 3). In Ca^2+^-free Krebs-Henseleit solution (contained 0.5 mM EGTA), SM extract-induced contraction of LES was significantly different from the contractile response in Krebs-Henseleit solution.

Smooth muscle contraction of LES is generally related to an increase in cytosolic Ca^2+^ concentration which is produced by trans-membrane Ca^2+^ entry and/or by intracellular Ca^2+^ release [18]. Thus, in this *ex vivo* contraction study of LES in rats, these above results showed the extracellular Ca^2+^ influx pathway other than the L-type Ca^2+^ channel may be the main pathway of SM extract-induced contraction of LES. There are several possible pathways for calcium entrance other than the L-type Ca^2+^ channel in SM extract-induced smooth muscle contraction of LES. The results could be contributed to agonist-induced contraction as well as tone, including capacitative Ca^2+^ entry [21], a T-type Ca^2+^ channel [22] and other sarcolemmal Ca^2+^ channels, such as nonselective cationic channels and receptor-operated calcium channels [23].

### 2.7. Effects of SM Extracts on Cytosolic Concentration of Free Ca^2+^ ([Ca^2+^]_i_) in Primary LES Smooth Muscle Cells

**Figure 4:**
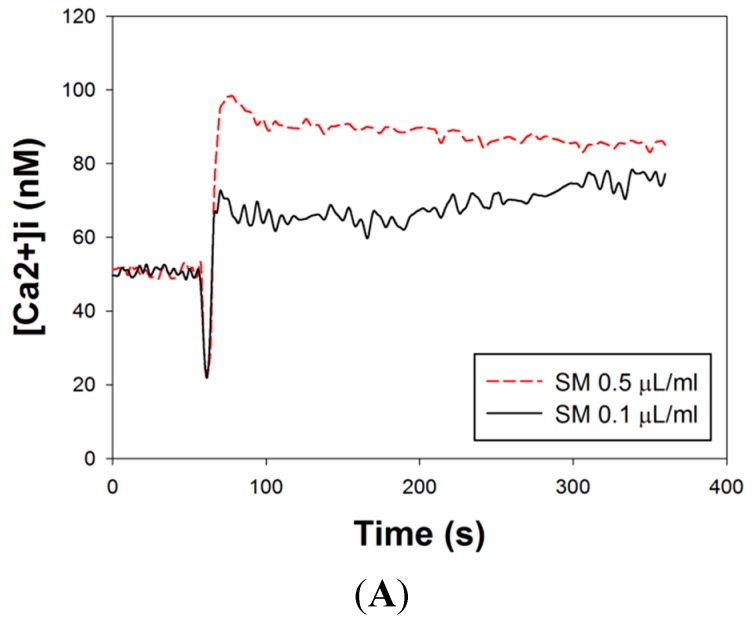

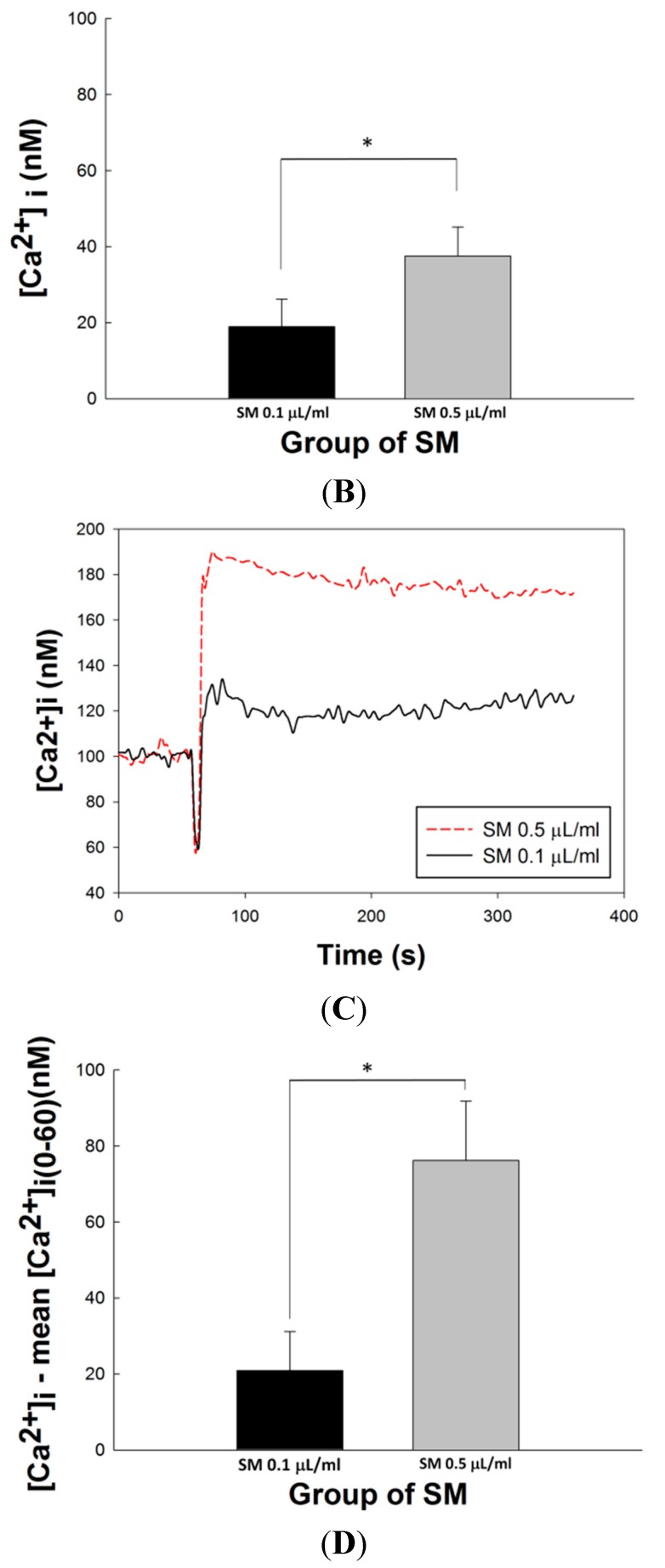
Effects of SM extracts on Ca^2+^ signaling in primary LES cells. SM extracts were diluted as 0.5 μL/mL or 0.1 μL/mL in Ca^2+^-containing or Ca^2+^-free medium. The SM extracts were added at the 60th s (**A**) Mean concentration-response plots of SM-induced Ca^2+^ signals in Ca^2+^-free medium; (**B**) The mean ([Ca^2+^]_i(61–360)_ − mean [Ca^2+^]_i(0–60)_) in primary LES cells in Ca^2+^-free medium during the interval of 61–360 s after SM (0.5 μL/mL or 0.1 μL/mL) treatment; (**C**) Mean concentration-response plots of SM-induced Ca^2+^ signals in Ca^2+^-containing medium; (**D**) The mean ([Ca^2+^]_i(61–360)_ − mean [Ca^2+^]_i(0–60)_) in primary LES cells in Ca^2+^-containing medium during the interval of 61–360 s after SM (0.5 μL/mL or 0.1 μL/mL) treatment. * Significantly difference between these two groups at *p* < 0.05.

The effects of SM extracts on [Ca^2+^]_i_ flow from extracellular entry or intracellular release were investigated in primary LES smooth muscle cells. The mean concentration-response plots of SM-induced increased [Ca^2+^]_i_ signals in Ca^2+^-free medium was shown in Figure 4A. SM extracts were added to primary LES smooth muscle cells at the 60th s (mean [Ca^2+^]_i_: 47.03 ± 13.43 nM at the 60th s). After addition of SM extracts, a short decay of [Ca^2+^]_i_ was noted and rapid rise of [Ca^2+^]_i_ followed. A sustained [Ca^2+^]_i_ was noted after [Ca^2+^]_i_ reached the top. The changes of [Ca^2+^]_i(61–360)_ were that mean [Ca^2+^]_i_ during 0–60 s as a background was subtracted from [Ca^2+^]_i_ at each time point during 61–360 s As showed in Figure 4B, the average of ([Ca^2+^]_i(61–360)_ − mean [Ca^2+^]_i(0–60)_) was calculated and the results in the groups of high (0.5 μL/mL) and low (0.1 μL/mL) concentration of SM extracts were 37.53 ± 8.83 nM and 18.95 ± 7.20 nM, respectively (*p* < 0.05, *n =* 3). This result indicated SM extracts induced intracellular Ca^2+^ release from intracellular stores (sarcoplasmic reticulum) in a dose-response manner.

The mean concentration-response plots of SM-induced increased [Ca^2+^]_i_ signals in Ca^2+^-containing medium are shown in Figure 4C. SM extract was added to primary LES smooth muscle cells at the 60th s (mean [Ca^2+^]_i_: 99.11 ± 4.61 nM at the 60th s). After addition of SM extract, [Ca^2+^]_i_ had a similar presentation as that in Ca^2+^-free medium. As shown in Figure 4D, the average of ([Ca^2+^]_i(61–360__)_ − mean [Ca^2+^]_i(0–60)_) was calculated and the results in the groups of high (0.5 μL/mL) and low (0.1 μL/mL) concentration of SM extracts were 76.15 ± 15.65 nM and 20.91 ± 10.26 nM, respectively (*p* < 0.05, *n =* 3). Comparing Figure 4D with Figure 4B, the averages of ([Ca^2+^]_i(61–360)_ − mean [Ca^2+^]_i(0–60)_) of these groups stimulated by high and low concentration of SM extract in the Ca^2+^-containing medium were significantly higher than those in the Ca^2+^-free medium, respectively (76.15 ± 15.65 *vs.* 37.53 ± 8.83 nM, 20.91 ± 10.26 *vs.* 18.95 ± 7.20 nM, both *p* < 0.05). Compared to low concentration SM extract, high concentrations of SM extract induce 3.64- and 1.98-fold increases (76.15 ± 15.65/20.91 ± 10.26 *vs.* 37.53 ± 8.83/18.95 ± 7.20) in Ca^2+^-containing (Figure 4D) and Ca^2+^-free (Figure 4B) medium, respectively (*p* < 0.05). These results indicate SM extracts induce extracellular Ca^2+^ entry into smooth muscle cells in addition to intracellular Ca^2+^ release in a dose-response manner.

### 2.8. Effect of SM Extracts on Confocal Images of Changes in Intracellular [Ca^2+^] in Primary LES Smooth Muscle Cells

**Figure 5:**
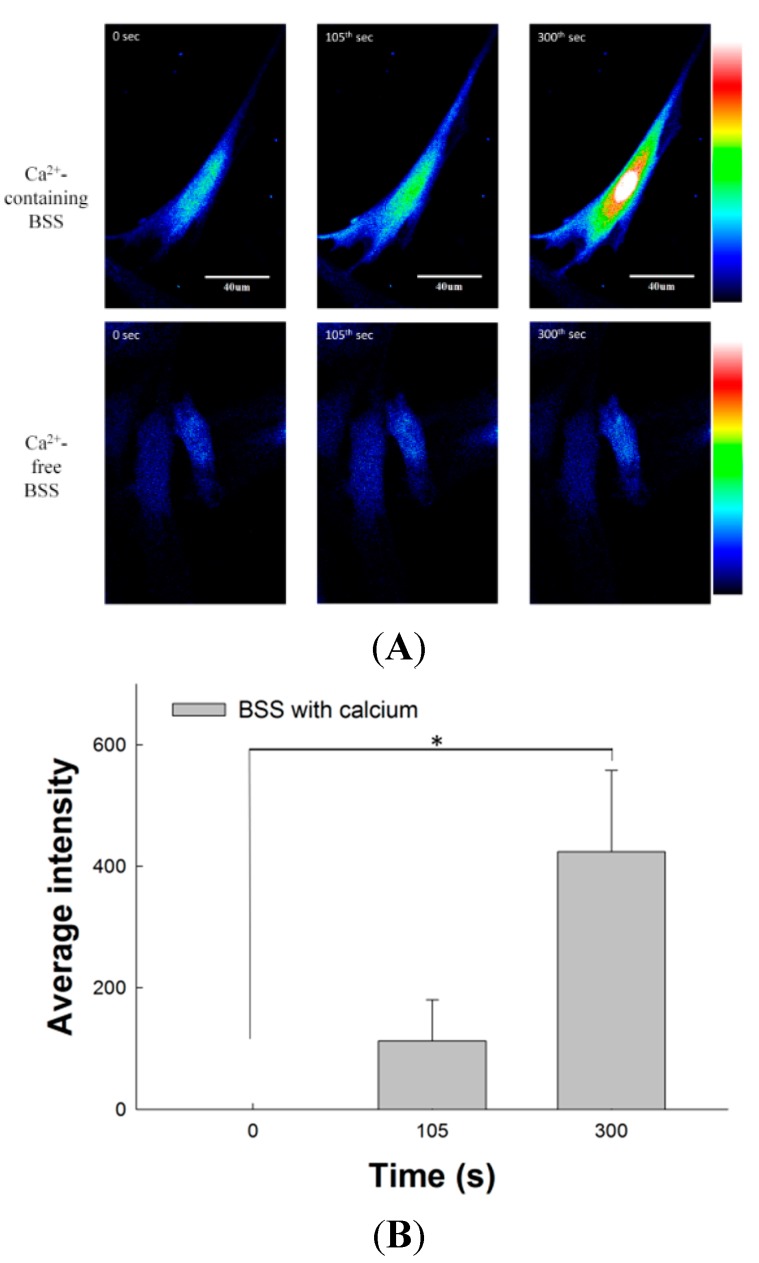

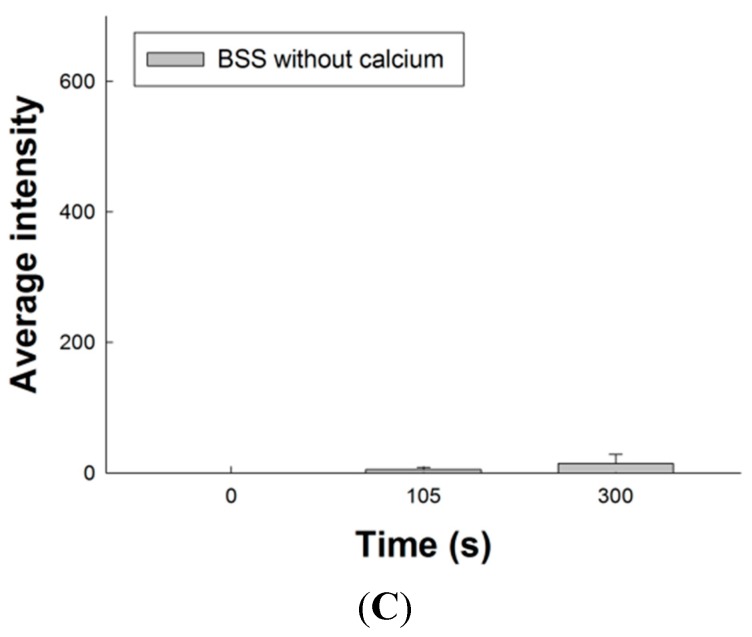
Effects of SM extracts on the fluorescence intensity of Ca^2+^ signaling in primary LES cells stained with fluo-4-acetoxymethyl ester and detected by confocal microscopy. SM extracts were diluted as 0.5 μL/mL in Ca^2+^-containing or Ca^2+^-free BSS. The SM extracts were added at the 60th s (**A**) The three images of upper row show the fluorescence intensity of Ca^2+^ signaling in primary LES smooth muscle cells increased significantly in the Ca^2+^-containing BSS after SM treatment. On the contrary, there was no significant change in the Ca^2+^-free BSS after SM treatment; (**B**) The mean relative fluorescence intensity (F − F_0_, where F_0_ = the first image of the sequence and F = subsequent images) in smooth muscle cells at the 300th s in the Ca^2+^-containing BSS after SM treatment increased significantly; (**C**) The mean relative fluorescence intensity in smooth muscle cells at the 105th or 300th s in the Ca^2+^-free BSS after SM treatment did not increase significantly. Scale bar represents 40 μm. * Significant difference between these two groups at *p* < 0.05.

Figure 5A shows the fluorescence intensity related to intracellular [Ca^2+^] in primary LES cells by confocal microscopy. The three images of the upper row were detected in Ca^2+^-containing Buffer Salt Saline (BSS). After stimulation by SM extract at the 60th s, the fluorescence intensity of intracellular [Ca^2+^] increased gradually from the 105th s (green) to the 300th s (red and bright). On the contrary, the fluorescence images of primary LES cells after SM treatment in Ca^2+^-free BSS showed a mild increase at the 105th s and had only a little yellow particle on central part at the 300th s Figure 5B showed the change of relative fluorescence intensity (F − F_0_, where F_0_ = the first image of the sequence and F = subsequent images) in the Ca^2+^-containing BSS. The relative fluorescence intensity (423.82 ± 133.68) of intracellular [Ca^2+^] in primary LES cells at the 300th s was significantly higher than that at 0 s (*p* < 0.05, *n* = 3). Figure 5C shows the change in relative fluorescence intensity (F − F_0_) in Ca^2+^-free BSS. The change of relative fluorescence intensity from 0 s, and 105th s to 300th s was at a relatively low level. These results indicate SM extracts induce extracellular Ca^2+^ entry into smooth muscle cells and increase fluorescence intensity. These *in vitro* findings are in good agreement with our results obtained in the *ex vivo* study, which shows SM-induced contraction of LES is mainly associated with extracellular Ca^2+^ influx.

**Figure 6:**
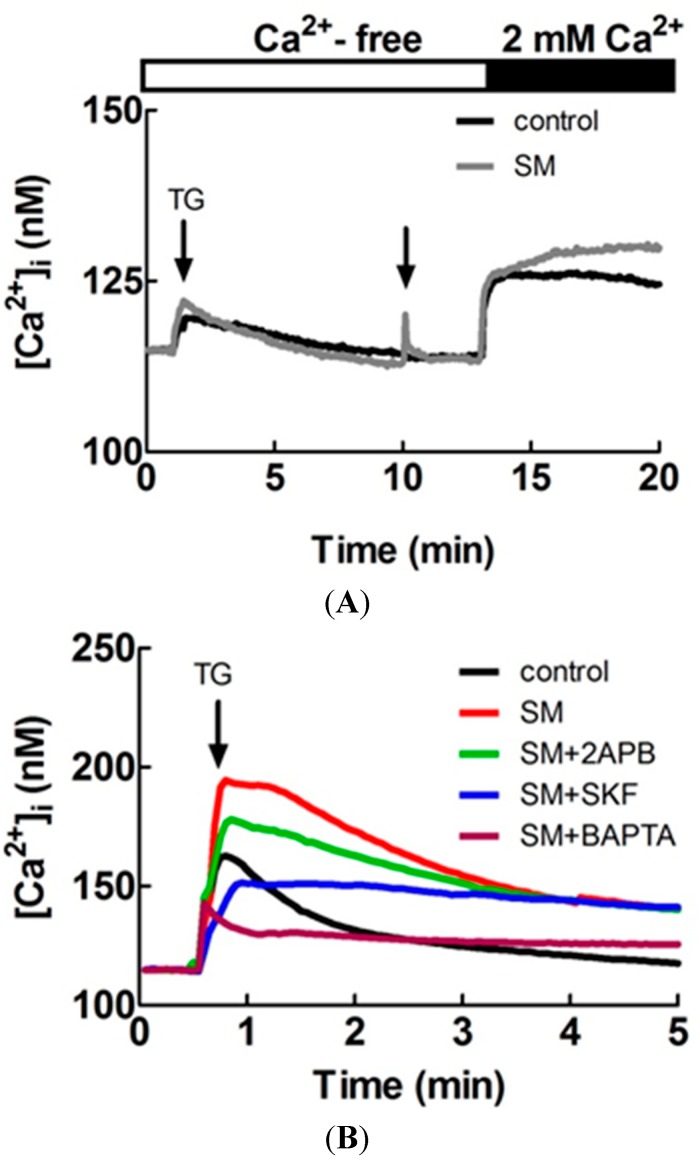
Effects of SM extracts on cytosolic concentration of free Ca^2+^ ([Ca^2+^]_i_) in primary LES cells stained with fluo-4 acetoxymethyl ester and detected by the Olympus CellˆR. (**A**) 1 μM thapsigargin (TG) treatment was performed at the 30th s and 0.5 μL SM extracts stimulations or Ca^2+^-free BSS solution (control group) was added to 1 mL Ca^2+^-free BSS solution at the 10th min. The [Ca^2+^]_i_ in the SM extract stimulation group was more than the control group after addition of 2 mM CaCl_2_ at the 13th min (*n =* 3); (**B**) Primary LES smooth muscle cells were pretreated with 0.5 μL SM extracts, 0.5 μL SM extract with 20 μM 2-aminoethoxydiphenyl borate (2APB), 0.5 μL SM extracts with 2-aminoethoxydiphenyl borate (2APB), 0.5 μL SM extracts with 20 μM SKF-96365 (SKF), 0.5 μL SM extracts with 25 μM 1,2-bis(2-aminophenoxy)ethane-*N*,*N*,*N′*,*Nʹ*-tetraacetic acid tetrakis(acetoxymethyl ester) (BAPTA-AM), and 0.5 μL BSS (control group) to 1 mL Ca^2+^-containing BSS solution. 1 μM TG was applied to induce Ca^2+^ influx at the 30th s The [Ca^2+^]_i_ in the group of only SM extracts pretreatment was more than the other groups (*n =* 3).

Figure 6A shows primary LES cells treated by 1 μM thapsigargin (TG) at the 30th s in calcium-free BSS solution and 0.5 μL SM extract stimulation or Ca^2+^-free BSS solution (control group) added to 1 mL Ca^2+^-free BSS at the 10th min. The [Ca^2+^]_i_ in the SM extract stimulation group was more than in the control group after addition of 2 mM CaCl_2_ at the 13th min (*n =* 3). These results indicate SM extracts increase [Ca^2+^]_i_ in primary LES cells by inducing extracellular Ca^2+^ entry into smooth muscle cells. Figure 6B shows primary LES smooth muscle cells pretreated with only 0.5 μL SM extracts, 0.5 μL SM extracs with 25 μM 2-aminoethoxydiphenyl borate (2APB, a store operated channel blocker), 0.5 μL SM extracts with 20 μM SKF-96365 (SKF, a store operated channel blocker), 0.5 μL SM extracts with 25 μM 1,2-bis(2-aminophenoxy)ethane-*N*,*N*,*N′*,*N′*-tetraacetic acid tetrakis (acetoxymethyl ester) (BAPTA-AM, a chelator of cytosolic Ca²^+^), and Ca^2+^-containing BSS (control group) to 1 mL Ca^2+^-containing BSS and 1 μM TG was applied to induce Ca^2+^ influx at the 30th s by depleting the endoplasmic reticulum calcium stores. The [Ca^2+^]_i_ in the group of only SM extracts pretreatment was more than the other groups (*n =* 3). These results indicate Ca^2+^ influx is the main pathway of increased [Ca^2+^]_i_ induced by SM extracts.

## 3. Experimental Section

### 3.1. Materials

This study was carried out in strict accordance with the recommendations in the Guide for the Care and Use of Laboratory Animals of Council of Agriculture, Executive Yuan, Taiwan. The protocol was approved by the Animal Care and Use Committee at E-Da Hospital (Permit Number: IACUC-103025). All rats were directly sacrificed by CO_2_ and all efforts were made to minimize suffering. Male Sprague-Dawley rats were obtained from the BioLASCO Taiwan (Taipei, Taiwan). Carbachol, atropine, nifedipine, papain, collagenase II, dispase II and buffer reagents were obtained from Sigma-Aldrich (St. Louis, MO, USA). Fura-2-acetoxymethyl ester (Fura-2 AM) and fluo-4 AM were purchased from Invitrogen (Carlsbad, CA, USA). Tetrodotoxin was purchased from Tocris Cookson Inc. (Avonmouth Bristol, UK). TA was obtained from the National Institute for Control of Biological and Pharmaceutical Products of China (Beijing, China) and its purity was more than 98% by HPLC analysis.

### 3.2. Preparation of SM Extracts

Dried roots of SM were obtained from Winpower Biotech. Co., Ltd. (Kaohsiung, Taiwan). The authentication of SM roots was done on the basis of standards and methods provided as listed in Zhonghua Bencao [24]. A herbarium sample, coding number ISU-CMPB-0012, was maintained in the School of Chinese Medicine for Post-Baccalaureate, I-Shou University (Kaohsiung, Taiwan) for future reference. The dried roots of SM (100 g) were cut up in about 1 cm lengths and then ground using a pulverizer. 95% Ethanol was used to extract the roots of SM three times for 18 h. The ethanol extracts were combined and concentrated to 50 mL under vacuum. A membrane filter (0.45 μm) was used to filter the solutions which were then infused into the HPLC for analysis or used in the study of contraction of LES. In this present study, the extraction yield of TA from SM roots was determined as follows [25]:

(1) Extraction yield (ww)=Mass of tanshinone (in extracted solution)Mass of material (roots of SM) ×100%

### 3.3. HPLC Analysis of TA in SM Extracts

The chromatographic system consisted of a LC-10AT pump, a SIL-10AF autosampler and a SPD-10Avp UV-VIS detector (Shimadzu, Kyoto, Japan). A Purospher^®^ STAR RP-18 endcapped (250 mm × 4.6 mm i.d., 5 μm) (Merck, Darmstadt, Germany) was applied for the separation and temperature was controlled at 25 °C. The mobile phase was made up of 75% acetonitrile, 12.5% methanol and 12.5% water at volumetric ratios and pH was titrated to 3.0 with glacial acetic acid. The detection was monitored at 270 nm. The mobile phase was delivered at a rate of 1.0 mL/min and the volume of injection loop was 10 μL. Diazepam was used as the internal standard. The sample calibration curve for TA was linear (r = 0.9999) within the range 0–50 mg/mL. Intra- and inter-day coefficients of variation of the assays were less than 5% (*n =* 6) [26].

### 3.4. Measurement of Contraction of LES in Rat

Measurements of contraction of the isolated LES were performed according to the procedure previously described in detail [16,27]. Sprague-Dawley (SD) rats, each weighing 350–400 g, were euthanized with CO_2_ directly in a sealed plastic bag and confirmed to be dead through observation of discontinuation of respiration and palpation of cessation of heartbeat. The abdomen was opened by surgical scissors and the stomach and lower portion of the esophagus were removed and cut open in the longitudinal direction along the greater curvature. The mucosa was cleared away by surgical forceps. A transverse strip (10 mm long and 2 mm wide) was cut from the LES, which was identified as a pink thickened muscle between the stomach and the esophagus. The muscle strips were mounted in 7-mL organ baths containing Krebs-Henseleit solution (118 mM NaCl, 25 mM NaHCO_3_, 4.7 mM KCl, 14 mM glucose, 1.2 mM NaH_2_PO_4_, and 1.8 mM CaCl_2_) at 37 °C and being oxygenated with 95% O_2_ + 5% CO_2_. The muscle strips were linked by surgical silk sutures to isometric transducers (FT.03; Grass Technologies, West Warwick, RI, USA), which were attached to an amplifier (Gould Instrument Systems, Valley View, OH, USA) and a computer recording system (BIOPAC Systems, Santa Barbara, CA, USA). The resting tension of the muscle strips was adjusted to 1.0 g. After a 30-min equilibration period, carbachol was added into organ bath to check the activity of muscle strip and then washed out. Another equilibration period was performed. SM extracts (40 μL, 100 μL, 180 μL, and 280 μL) were added in a cumulative-dose administration into 7-mL organ baths. The muscle strips of LES were removed from the bath, blotted dry, and weighed (tissue wet weight) at the end of each experiment. The AUC (g/g tissue/s) was calculated.

### 3.5. Effect of Tetrodotoxin, Atropine, Nifedipine and Ca^2+^-Free Krebs-Henseleit Solution with EGTA on SM Extract-Induced Contraction of LES in Rat

To investigate the mechanism of SM extract-induced contraction of LES, the isolated LES was pretreated with the Na^+^ channel blocker TTX (10^−6^ M) and followed 15 min later by addition of SM extract to the organ bath [28,29]. The isolated LES was also pre-treated with the non-selective muscarinic receptor antagonist atropine (10^−6^ M) followed 6 min later by addition of SM extract. Isolated LES was also pretreated with the L-type Ca^2+^ channel blocker nifedipine (10^−6^ M) followed 20 min later by addition of SM extract. Ca^2+^-free Krebs-Henseleit solution had a similar composition of Krebs-Henseleit solution except for the Ca^2+^ and it contained 0.5 mM EGTA. The solution in organ bath was replaced by Ca^2+^-free Krebs-Henseleit solution with EGTA and SM extracts were added into organ baths [16,30].

### 3.6. Enzymatic Isolation of Primary LES Smooth Muscle Cells

Single smooth muscle cells from LES of Male SD rats were freshly isolated using enzymatic digestion. Briefly, muscular strips of the LES were cut in small pieces and incubated in a 30 μL/mL papain solution for 10 min, washed with Hank's Balanced Salt Solution (HBSS) and incubated for an additional 15 min with a mixture of collagenase type II 4 mg/mL and dispase II 4.5 mg/mL at 37 °C, then washed with HBSS again. After stopping the enzyme reaction with Dulbecco’s Modified Eagle medium (DMEM), the cells were cultured in dishes [31].

### 3.7. [Ca^2+^]_i_ Measurements

Fura-2 AM was applied as a Ca^2+^ probe to detect the [Ca^2+^]_i_ and measurement of [Ca^2+^]_i_ was performed according to the procedure previously described in detail [32,33]. 10^−6^ M AM form of fura-2 was added to trypsinized cells (10^6^/mL) at 25 °C for 30 min in DMEM. Fura-2 fluorescence measurements were performed in a water-jacketed cuvette (25 °C) with continuous stirring; the cuvette contained 1 mL of medium and 0.5 million cells. A Shimadzu RF-5301PC spectrofluorophotometer (Shimadzu, Kyoto, Japan) was used to monitor fluorescence by recording excitation signals at 340 nm and 380 nm of light and emission signal at 510 nm of light at 2 s intervals. 1% Triton X100 and 20 mM EGTA were sequentially added at the end of each experiment to determine maximum and minimum fluorescence values. Ca^2+^-containing medium is composed of 5 mM KCl, 140 mM NaCl , 2 mM CaCl_2_, 1 mM MgCl_2_, 10 mM HEPES, and 5 mM glucose; the pH was adjusted to 7.4 with 1 N NaOH. Ca^2+^-free medium had a similar composition except for the absence of Ca^2+^ and it contained 1 mM EGTA. The extracts of SM (0.5 μL or 0.1 μL) were added into 1 mL Ca^2+^-containing or Ca^2+^-free media at the 60th s to measure the Ca^2+^ levels in primary LES cells.

### 3.8. Confocal Microscope Fluorescent Images

Fluo-4 AM was applied as Ca^2+^-sensitive fluorescent indicator and the fluorescence intensity of intracellular [Ca^2+^] in primary LES smooth muscle cells were detected using a confocal microscope (Olympus FV 1000, Tokyo, Japan). Briefly, 10^5^/mL primary LES smooth muscle cells were seeded onto the glass coverslips in six well plastic plates to grow. Two days later, culture medium was removed and cells were washed with BSS and incubated with 1 μM fluo-4 AM solution for 20 min at 37 °C. Fluo-4 AM solution was removed and cells was washed out again with BSS. The coverslip was nipped on special holder and the confocal microscope was used to detect calcium imaging at 3 s intervals for 6 min. The 0.5 μL extracts of SM were sequentially added into 1 mL Ca^2+^-containing or Ca^2+^-free BSS on the holder at 1st min. BSS consisted of 5.4 mM KCl, 5.5 mM D-glucose, 1 mM MgSO_4_, 130 mM NaCl, 20 mM HEPES, 2 mM CaCl_2_ and pH was adjusted to 7.4. Ca^2+^-free BSS had a similar composition except Ca^2+^. Similarly, CellˆR IX81 fluorescence microscope (Olympus, Hamburg, Germany) with fluo-4 AM staining was also applied to detect the intracellular calcium responses to stimulation, such as by CaCl_2_ or TG. Calcium concentration was calculated using the following formula: [Ca^2+^]_i_ = K_D_ × [(F − F_min_)/(F_max_ − F)]. Plotting the fluorescence intensity *versus* [Ca^2+^]_i_ yielded the calibration curve with the formula of: [Ca^2+^]_i_ = K_D_ × [(F − F_min_)/(F_max_ − F)], where K_D_ = 150.5 nM, F = Fluo-4 intensity, F_max_ = 640, and F_min_ = 21.7 for Fluo-4 [34,35].

### 3.9. Data Analysis

These data were expressed as means ± standard deviation, and statistical analysis of the results was performed by using Student *t* test. In all cases, differences were considered significant when *p* < 0.05.

## 4. Conclusions

The present study demonstrates that SM causes tonic contraction of LES. The SM-induced contraction may not relate to neuronal conduction, muscarinic receptors, and L-type voltage-dependent calcium channels. This study provides the evidence that the SM-induced contraction of LES is mainly associated with extracellular Ca^2+^ influx, especially through store operated calcium channels. Hence, SM has the potential to treat the patients with proton pump inhibitor refractory GERD.
